# Supplementary figures and images for: Neutrophil degranulation, NETosis and platelet degranulation pathway genes are co-induced in whole blood up to six months before tuberculosis diagnosis
Source: PLoS One. 2022 Dec 1;17(12):e0278295. doi: 10.1371/journal.pone.0278295 (PMC9714760; doi:10.1371/journal.pone.0278295)

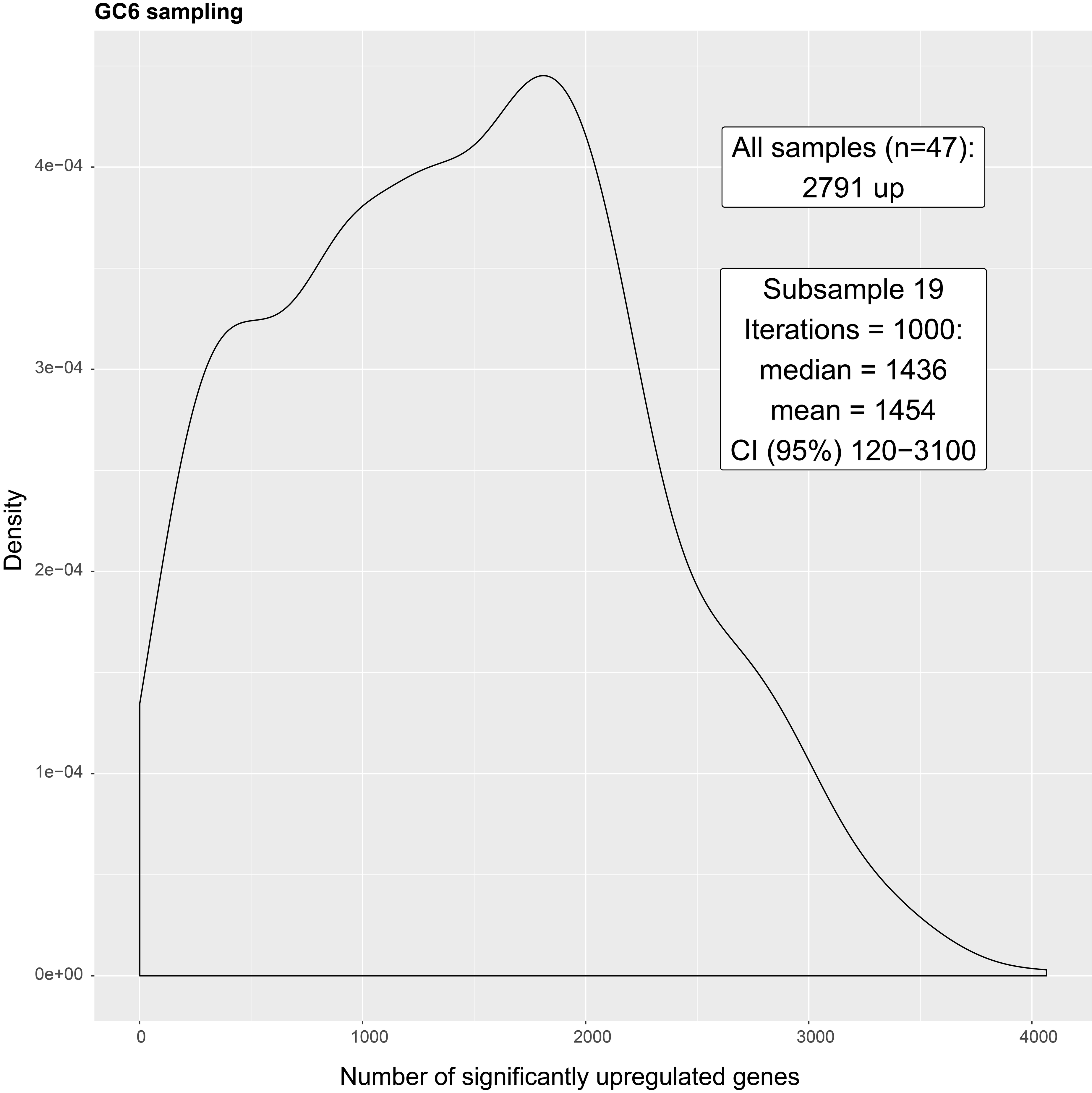

Supplement: S1 Fig — Subsampling was performed on the 1–6 month-before TB group to investigate the effect the larger sample size (n = 47) had on the number of significantly up-regulated genes identified. A total of 1000 differential expression analysis tests were performed using a sample size of 19 which is similar to the earlier 12 month before (n = 19) and 18-month before time points (n = 18). The mean number of significantly up-regulated genes identified in the subsampling was 1,436 (95% CI:120, 3,100). This number was lower than the 2,791 identified with the full 47 samples but far higher than the number identified in the 18 (249; n = 19) and 12 (492; n = 18) months before diagnosis groups. (TIF) [file pone.0278295.s006.tif]

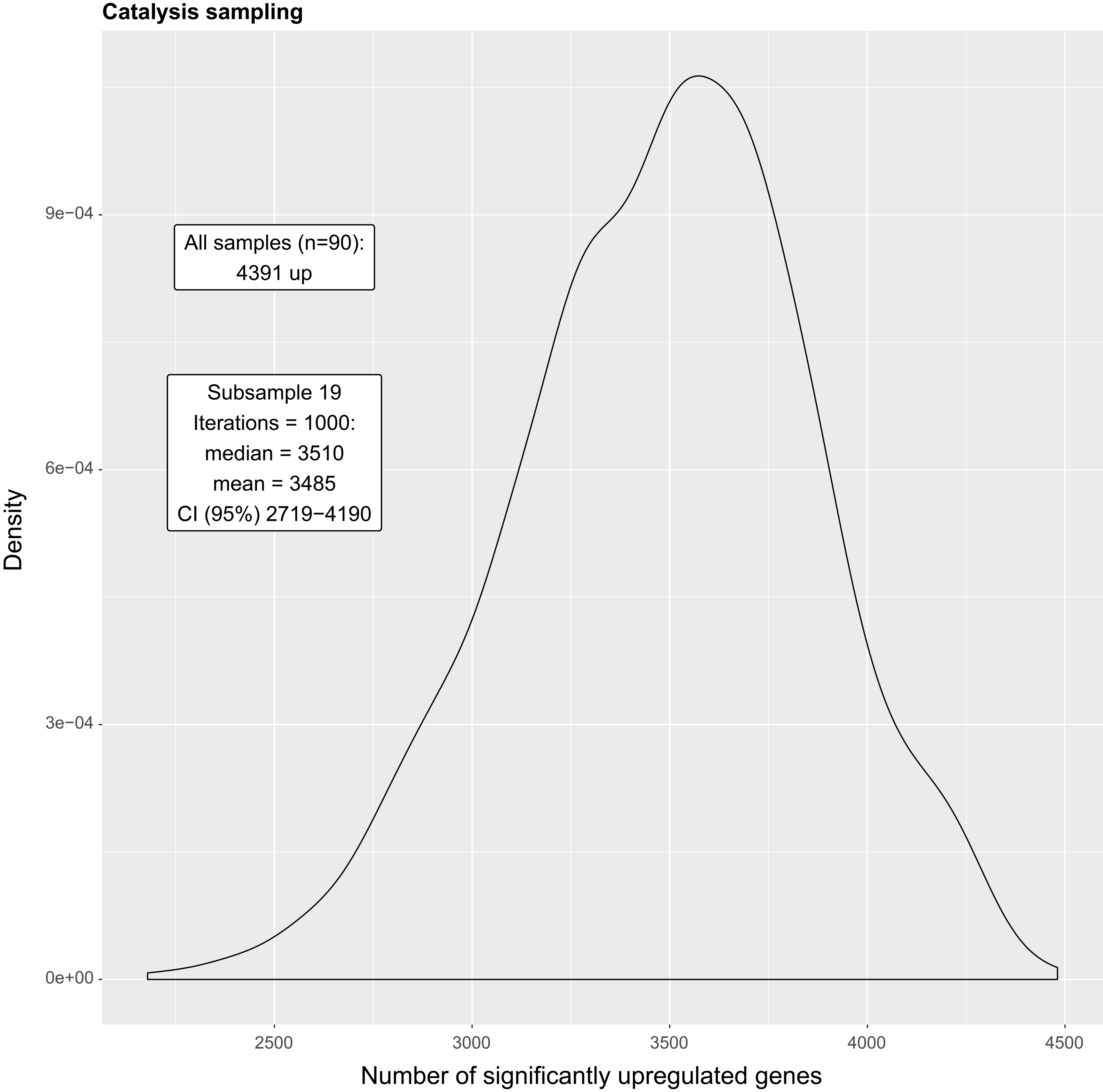

Supplement: S2 Fig — Subsampling was performed on the Catalysis study TB group to investigate the effect the larger sample size (n = 90) had on the number of significantly up-regulated genes identified. A total of 1000 differential expression analysis tests were performed using a sample size of 19 which is similar to the earlier 12 month before (n = 19) and 18-month before time points (n = 18). The mean number of significantly upregulated genes identified in the subsampling was 3,485 (95% CI:2,719, 4,190). This was lower than the 4,391 identified with the full 90 samples but far higher than the number identified in the 18 (249; n = 19) and 12 (492; n = 18) months before diagnosis groups. (TIF) [file pone.0278295.s007.tif]
