# Supplementary material for: Neutrophil degranulation, NETosis and platelet degranulation pathway genes are co-induced in whole blood up to six months before tuberculosis diagnosis
Source: PLoS One. 2022 Dec 1;17(12):e0278295. doi: 10.1371/journal.pone.0278295 (PMC9714760; doi:10.1371/journal.pone.0278295)
Supplement: Attachment — Submitted filename: Meier_NeutrophilTranscriptomics_ResponseToReview.docx [file pone.0278295.s008.docx]

**COMMENTS FROM EDITORIAL TEAM:**

When submitting your revision, we need you to address these additional requirements.

1. Please ensure that your manuscript meets PLOS ONE's style requirements, including those for file naming. The PLOS ONE style templates can be found at
   <https://journals.plos.org/plosone/s/file?id=wjVg/PLOSOne_formatting_sample_main_body.pdf>

and

<https://journals.plos.org/plosone/s/file?id=ba62/PLOSOne_formatting_sample_title_authors_affiliations.pdf>

**RESPONSE:**

*We have checked the style requirements documents and used the Word Template. We have ensured that the formatting was not altered from the format in the template.*

2. We note that you have included the phrase “data not shown” in your manuscript. Unfortunately, this does not meet our data sharing requirements. PLOS does not permit references to inaccessible data. We require that authors provide all relevant data within the paper, Supporting Information files, or in an acceptable, public repository. Please add a citation to support this phrase or upload the data that corresponds with these findings to a stable repository (such as Figshare or Dryad) and provide and URLs, DOIs, or accession numbers that may be used to access these data. Or, if the data are not a core part of the research being presented in your study, we ask that you remove the phrase that refers to these data.

**RESPONSE:**

*The phrase “data not shown” has been removed and the paragraph updated to the following:*

*Mapping statistics revealed that for the GC6-74 cohort,* *a mean of 47.5 million reads [(47.1, 47.9: 95% CI; 85.9% (95% CI: 85.7, 86.1)] mapped uniquely to the human genome with 87.8 % (95% CI: 87.7, 87.9) of these mapping to genes. For the Catalysis cohort, a mean of 40.1 million reads [(39.4, 40.8: 95% CI; 88.6% (88.3, 88.9: 95% CI)] mapped uniquely to the human genome with 88.8 % (95% CI: 88.7, 88.9) mapping to genes.*

3. Please include your full ethics statement in the ‘Methods’ section of your manuscript file. In your statement, please include the full name of the IRB or ethics committee who approved or waived your study, as well as whether or not you obtained informed written or verbal consent. If consent was waived for your study, please include this information in your statement as well.

**RESPONSE:**

We used data deposited in public repositories that had previously been collected with ethical approval and informed consent. In the revised submission, we now list all the prior ethics approvals under which the data were collected.

**In paper:**

*Further details of the study, including ethical approval details have been described previously [23].*

*For the GC6-74 study [23] the reported ethics approvals are as follows (from one of the appendices):*

| ***Site*** | ***Ethics Review Committee Protocol*** | ***no.*** |
| --- | --- | --- |
| *SUN* | *Stellenbosch University Institutional Review Board* | *N05/11/187* |
| *MRC* | *Joint Medical Research Council and Gambian Government* | *SCC.1141vs2* |
| *MAK* | *Uganda National Council for Science and Technology* | *MV 715* |
| *MAK* | *University Hospitals Case Medical Centre* | *12-95-08* |
| *AHRI* | *Armauer Hansen Research Institute (AHRI)/All Africa Leprosy, TB and Rehabilitation Training Center (ALERT)* | *P015/10* |
| *UCT* | *(GC6) University of Cape Town Human Research Ethics Committee HREC* | *013/2013* |

*For the Catalysis study [28], the ethical approval (single site) was as follows:*

*SU	Stellenbosch University Institutional Review Board	N10/01/013*

We have inserted the following in the Methods section at the end of the sub-section titled “Study outline and data sources”

*Both source studies (the GC6-74 study and the Catalysis TB Biomarker study) were performed with ethical approvals and required written informed consent. For the GC6-74 study the following ethics approvals applied (as described in [23]): Stellenbosch University, South Africa,* *Stellenbosch University Institutional Review Board, N05/11/187; UK Medical Research Council Unit, the Gambia, Joint Medical Research Council and Gambian Government, SCC.1141vs2; Makarere University, Uganda, Uganda National Council for Science and Technology, MV 715, and University Hospitals Case Medical Centre, 12-95-08; Armauer Hansen Research Institute, Ethiopia, Armauer Hansen Research Institute (AHRI)/All Africa Leprosy, TB and Rehabilitation Training Center (ALERT), P015/10; and the University of Cape Town, South Africa, University of Cape Town Human Research Ethics Committee (HREC), 013/2013. For the Catalysis TB Biomarker study, the ethical approval was from the Stellenbosch University, South Africa, Stellenbosch University Institutional Review Board, N10/01/013 [28].*

4. Please review your reference list to ensure that it is complete and correct. If you have cited papers that have been retracted, please include the rationale for doing so in the manuscript text, or remove these references and replace them with relevant current references. Any changes to the reference list should be mentioned in the rebuttal letter that accompanies your revised manuscript. If you need to cite a retracted article, indicate the article’s retracted status in the References list and also include a citation and full reference for the retraction notice.

**RESPONSE**:

*We performed a thorough review of all publications cited (on October 2, 2022). None of the citations were retracted. There were however two corrections (but these are indicated as such on the publisher site and are corrected in the online published versions), and 19 comments (indicated on the publisher web site and on PubMed).*

**COMMENTS FROM REVIEWER** Arshad Khan

This manuscript by Stuart Meier et al, is a well designed and well conceived study and identifies some of the key host signatures of early diagnosis of TB. Through RNAseq analysis of whole blood and PET-CT imaging in 2 different cohorts spanning early stage of infection to TB, the study provides key insights into the probable pathways that could be engaged in TB pathogenesis during progression of the disease. Through this analysis, the authors show clear correlation between activation of neutrophils, platelet degranulation and NET formation, and progression of disease over a period of 18 months. Overall the study has very high scientific merit and advances our understanding about the key components of the host that could be involved in TB immunopathogenesis.

**RESPONSE:**

*We thank the reviewer for the constructive comments, and outline responses to specific comments below.*

Some of the minor comments, if addressed can help improve this manuscript further:

1. One of the sub division of cohorts could be house hold contacts that developed TB vs those that never developed TB. If the analysis can include these sub cohorts, it can provide more comprehensive evidence of the claimed findings.

**RESPONSE:**

*The reviewer is correct that a purified set of controls of individuals resistant to TB (never developed TB) would be more powerful for detecting the difference between TB cases and controls. Unfortunately, the design of the GC6-74 study did not permit detecting such a sub-cohort. The analysis of the GC6-74 progressor cohort compared individuals that developed TB to those who did not during the period of 24 months of follow-up. The subjects in this study were all household contacts of recently diagnosed TB cases. Subjects who developed TB after 3 months (washout) and within two years of enrollment in the study were classified as TB progressors while those who did not were the control group. Most cases developed TB within a year with a median of 9 months and an IQR (6,17). Determining that a sub-cohort of the 4,362 subjects who did not develop TB by 24 months never developed TB would be an enormous task both in terms of labor and cost, and would be extremely difficult in the high prevalence of TB in each of the regions from which the cohort was derived.*

*The potential of undetected TB, either latent or incipient, among the controls, would however only decrease the power to detect the effect and not fundamentally alter the observations. One could phrase it to say that the observations are more robust since the controls were not highly selected. Certainly, more selected, or purified, controls could have provided insight on aspects of the analysis that could have been obscured by the potential presence of disease signals among the controls.*

We have added the following sentence at the end of the first paragraph under the Study groups and data quality sub-section of Results.

*Due to the finite follow-up some of the subjects classified as non-progressor might have had pre-clinical TB. Such subjects in the “control” group would dilute the disease signals, possibly obscuring some insights, but would not alter the fundamental findings.*

2. It is not clear from this manuscript if PET-CT imaging of TB progresser's was done and compared with house-hold contacts that never developed TB. Perhaps a PET-CT from this cohort can be further divided into LTBI positive vs LTBI negative to better predict if PET-CT data hold any true relevance.

**RESPONSE:**

*The PET-CT imaging was only performed on the Catalysis TB cohort (a longitudinal treatment response study). Unfortunately, it was not available for the GC6 progressor cohort (a prospective longitudinal follow-up study).*

3. The discussion can provide more insight about the relationship between Type 1 IFN signaling and Neutrophil activation/NETOsis pathways.

**RESPONSE:**

*Thank you for this important comment. We have revised the relevant paragraph in the Discussion about type I IFN, as below.*

*Submitted manuscript:*

*Given the early and sustained level of type I IFN signaling observed during TB progression, it is interesting to note that IFN-α can prime neutrophils for NETosis [58]. In turn, NETs can stimulate type I IFN production in plasmacytoid dendritic cells (pDCs), prolonging the inflammatory response [59].*

*Revised manuscript:*

*Given the early and sustained level of type I IFN signaling observed during TB progression, it is interesting to note that in TB susceptible mice, type I IFN signaling has been shown to induce NETosis through activation of interferon α and β receptor subunit 1, which is associated with enhanced mycobacterial growth at infection sites and enhanced TB pathogenesis [58]. The same study identified NETs in nectrotic lung lesions of TB patients that responded poorly to treatment. Further, serum from patients with autoimmune disorders that have elevated levels of type I IFNs, as well as exogenous IFN-α, has been shown to stimulate neutrophil NET production in vitro suggesting that type I IFN prime neutrophils for NET production [59-61]. In turn, self-DNA and antimicrobial peptides released with NETs, have been reported to induce the chronic activation of plasmacytoid dendritic cells and secretion of type I IFNs in SLE patients creating a positive feedback loop that prolongs the inflammatory response [62]. The upregulated type I IFN signaling observed in this study that precedes neutrophil activation and NETosis, along with the above-mentioned studies, is consistent with type I IFN signaling diving neutrophil activation and NETosis during TB progression and enhancing TB pathogenesis.*

4. Primary TB can be infectious as has been shown in earlier studies, whereas authors here suggest otherwise (Line 387 discussion section).

**RESPONSE:**

*We thank the reviewer for detecting this inference that was not intended. We have revised the manuscript as below.*

*Submitted manuscript: (1^st^ para of discussion)*

*Our results revealed an early induction of IFN-related signaling at 18 months before diagnosis. A strong induction of neutrophil and platelet degranulation and NETosis related genes was detected 6 months before TB diagnosis and persisted in TB patients supporting a pathogenic role of these responses in disease development. Although these processes have previously been detected in severe TB, here we document that they occur well before disease diagnosis, indicating that they may be critical in mediating lung tissue destruction and progression from non-infectious latent or primary TB to contagious TB.*

*Revised manuscript:*

*Our results revealed an early induction of IFN-related signaling at 18 months before diagnosis. A strong induction of neutrophil and platelet degranulation and NETosis related genes was detected 6 months before TB diagnosis and persisted in TB patients supporting a pathogenic role of these responses in disease development.* *Although these processes have previously been detected in patients diagnosed with active TB, here we document that they occur well before diagnosis, indicating that they may be critical in mediating lung tissue destruction and progression from infection with M.tb to active TB.*

5. Figure 3 is completely blur and not readable at all.

**RESPONSE:**

*We apologize to the reviewer that the image quality in the review document was poor. It appears that the PLOS One submission system reduces the quality of the images, possibly to keep the size of the submitted document down to a reasonable magnitude. We agree that the quality of the figures as produced by the PLOS One online submission system is sub-optimal. The submitted figures were high-quality and we would be happy to share these with the reviewer. Perhaps the editorial system can provide the reviewer access to the original high-quality figures?*

**Additional Changes.**

During the revision we became aware that the PET status (PET-high and PET-low) was not in the published datasets or in a public repository. We therefore created an additional Supplementary document that has the PET status for the specimens used here connecting these with the Gene Expression Omnibus identifiers. These data are now presented in **S1 Table. PET category for the Catalysis study specimens**.
